# From form to function: the role of Nox4 in the cardiovascular system

**DOI:** 10.3389/fphys.2012.00412

**Published:** 2012-11-01

**Authors:** Feng Chen, Stephen Haigh, Scott Barman, David J. R. Fulton

**Affiliations:** ^1^Vascular Biology Center, Georgia Health Sciences UniversityAugusta, GA, USA; ^2^Department of Pharmacology and Toxicology, Georgia Health Sciences UniversityAugusta, GA, USA

**Keywords:** Nox4, NADPH oxidase, reactive oxygen species, H_2_O_2_, subcellular localization

## Abstract

The NADPH oxidase (Nox) family of proteins is comprised of seven members, including Noxes1–5 and the Duoxes 1 and 2. Nox4 is readily distinguished from the other Nox isoforms by its high level of expression in cardiovascular tissues and unique enzymatic properties. Nox4 is constitutively active and the amount of reactive oxygen species (ROS) contributed by Nox4 is primarily regulated at the transcriptional level although there is recent evidence for post-translational control. Nox4 emits a different pattern of ROS and its subcellular localizations, tissue distribution and influence over signaling pathways is different from the other Nox enzymes. Previous investigations have revealed that Nox4 is involved in oxygen sensing, vasomotor control, cellular proliferation, differentiation, migration, apoptosis, senescence, fibrosis, and angiogenesis. Elevated expression of Nox4 has been reported in a number of cardiovascular diseases, including atherosclerosis, pulmonary fibrosis, and hypertension, cardiac failure and ischemic stroke. However, many important questions remain regarding the functional significance of Nox4 in health and disease, including the role of Nox4 subcellular localization and its downstream targets. The goal of this review is to summarize the recent literature on the genetic and enzymatic regulation, subcellular localization, signaling pathways, and the role of Nox4 in cardiovascular disease states.

## Introduction

Reactive oxygen species (ROS) refer to a group of small reactive molecules that include superoxide (O^·−^_2_), hydrogen peroxide (H_2_O_2_), hydroxyl (·OH^−^), and hypochlorite (OCl^−·^) (Bedard and Krause, 2007). ROS are a consequence of aerobic metabolism and react avidly with other molecules, cellular lipids, proteins, and nucleic acids. Over time, cells have evolved strategies to regulate ROS levels to ensure the fidelity of physiological processes and survival. Low to moderate levels of ROS have been shown to contribute to important functions, such as cell differentiation, migration, adhesion, senescence, growth, and apoptosis. In contrast, a variety of diseases, such as cancer, neurological, and most relevant to this review, cardiovascular disease, are associated with elevated ROS levels (Lambeth, 2007; Lassegue and Griendling, 2010). A significant source of cellular ROS is the NADPH oxidases (Noxs) family of enzymes.

**Figure 1:**
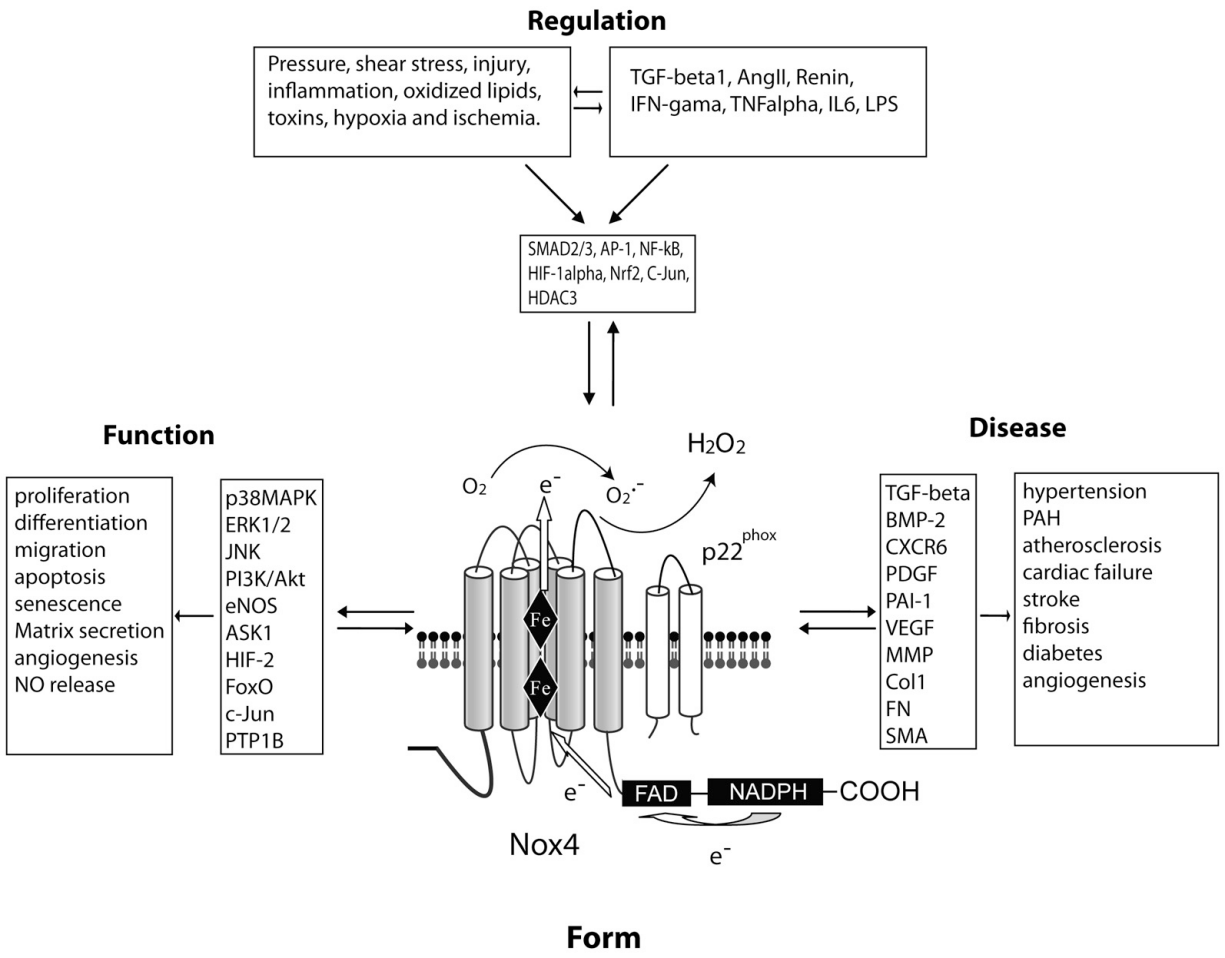
**A central role for Nox4 in the regulation of vascular cell function and cardiovascular disease.** FORM: Nox4 spans the membrane six times and produces hydrogen peroxide constitutively due to a histidine residue in the last transmembrane loop (E-loop). These enzymatic properties influence the FUNCTION of the cell via various signaling intermediaries. The expression level of Nox4 can be REGULATED via a number of stimuli and soluble factors which activate distinct transcription factors. These events have been proposed to influence various DISEASE processes.

All five Nox enzymes, including Nox4 are dual heme containing transmembrane oxidoreductases that span the membrane six times (see Figure 1). Catalytic activity originates with the binding of NADPH to a C-terminal site and the transfer of electrons through FAD to the two heme residues and, ultimately, to molecular oxygen to produce ROS. The mechanisms governing the activation of Noxes and production of ROS are different. The activation of Nox1–3 are analogous, and involves a combination of phosphorylation and protein–protein interactions. The subunits of Nox1–3 include p22phox, p40phox, p47phox, p67phox, NOXO1, and NOXA1 and the small G-proteins, Rac and Rap1a (Lassegue and Clempus, 2003; Griendling, 2004; Lassegue and Griendling, 2010). The cooperative binding of these subunits to the transmembrane Nox enzymes initiates electron transfer and is triggered by various stimuli including Angiotensin II. Nox5 does not require cytosolic or membrane subunits and has been reviewed in detail elsewhere (Fulton, 2009).

## Enzymatic properties of Nox4

Nox4 colocalizes with and directly binds the integral membrane protein p22phox which is necessary for Nox4 activity (Bedard and Krause, 2007) to stabilize p22phox expression (Martyn et al., 2006). The binding and activation of Nox4 by p22phox does not depend on the proline rich region of p22phox which is important in the regulation of Nox1–3 (Kawahara et al., 2005). A further distinction is that unlike Nox1–3, Nox4 does not require the binding of cytosolic proteins for ROS production (Martyn et al., 2006) and instead produces ROS constitutively. This is due to unique characteristics of the C-terminus of Nox4 that facilitates the constitutive transfer of electrons from NADPH to FAD (Nisimoto et al., 2010). Yet another distinguishing feature of Nox4 is the inability to detect O^·−^_2_ production in Nox4 transfected cells. Instead, robust production of H_2_O_2_ can be detected which contrasts from a mixture of O^·−^_2_ and H_2_O_2_ from Nox1–3 to Nox5 (Martyn et al., 2006; Serrander et al., 2007; Brandes et al., 2011). The mechanism underlying the preferential production of H_2_O_2_ vs. O^·−^_2_ is proposed to be a highly conserved histidine residue in the E-loop of Nox4 that promotes the rapid dismutation of O^·−^_2_ before it leaves the enzyme (Takac et al., 2011). Based on this information, prior reports of O^·−^_2_ production from endogenous Nox4 expressed in native cells are difficult to reconcile (Geiszt et al., 2000; Kuroda et al., 2005). It is likely that this issue stems from the methods used to detect different ROS and the concurrent expression of multiple Nox enzymes in native cells compared to the presence of unique or additional features of native cells than enable O^·−^_2_ production from Nox4. A further unique feature of Nox4 is its insensitivity to inhibitors of Hsp90 compared to the stimulus-dependent Nox enzymes. Nox1–3 and 5 interact with Hsp90 which facilitates O^·−^_2_ release and maintains protein stability (Chen et al., 2011). Hsp90 binds directly to the C-terminal region of Nox5 and binding to Nox1,3 is inferred from studies using chimeric proteins. Chimeric proteins that comprise the N-terminal region of Nox4 and the C-terminal region of Nox1 and 3 are sensitive to Hsp90 inhibitors. In contrast, fusion of the N-terminal region of Nox1 or Nox3 to the C-terminal region of Nox4 results in enzymes that are no longer sensitive to Hsp90 inhibition. Interestingly, these chimeric proteins produce only H_2_O_2_ suggesting that the C-terminal of Nox1–3 and 5 and chaperone-dependent folding are important for O^·−^_2_ release (Chen et al., 2011).

## Nox4 variants, expression, and genetic variation

The gene encoding Nox4 is found on chromosome 11 and contains 34 introns, and is transcribed into 16 spliced and 1 unspliced mRNAs. Of these variants, at least four splice variants encoding proteins have been reported in cells (Goyal et al., 2005). Only one of these variants was active as assessed by ROS production, but the mechanisms by which this variant generates ROS remain unclear as it lacks most of the transmembrane regions and part of the FAD binding region. Nox4 was originally identified in the kidney (Geiszt et al., 2000; Shiose et al., 2001) and in addition to cardiovascular tissues discussed below, it is expressed in hematopoietic stem cells (Piccoli et al., 2005), osteoclasts (Yang et al., 2001, 2004), keratinocytes (Chamulitrat et al., 2004), melanoma cells (Brar et al., 2002), neurons (Vallet et al., 2005; Kleinschnitz et al., 2010), adipocyte (Kanda et al., 2011), embryonic stem cells (Bartsch et al., 2011), chondrocytes (Grange et al., 2006; Kim et al., 2010), hepatic stellate cells (Ikeda et al., 2011), epithelial cells (Carnesecchi et al., 2011), and podocytes (Piwkowska et al., 2011). Using the human Nox4 standard gene sequence for comparison, approximately 2385 single nucleotide polymorphism (SNP) sites were found in the genomic DNA region of Nox4, and 45 SNP sites in the gene coding region. One of these sites conferred a frame shift mutation. The sequence variations described for Nox4 may affect gene replication or transcription, and possibly enzymatic function and ROS production. Therefore, it is possible they may contribute to the occurrence and development of disease. However, correlations between the genetic polymorphisms of Nox4 in different populations and disease related variations in Nox4 activity and or expression are poorly understood. Such associations would be aided by genetic studies investigating the relationship between Nox4 expression and function in diseases related to elevated ROS levels.

## Expression of Nox4 in blood vessels and the heart

The presence of Nox4 in vascular cells was documented soon after its initial discovery in murine and human kidney (Geiszt et al., 2000; Lambeth et al., 2000; Cheng et al., 2001; Shiose et al., 2001) and osteroclasts (Yang et al., 2001). Nox4 mRNA was found to be an abundant transcript in cultured rat and human aortic vascular smooth muscle cells (VSMC) (Lassegue et al., 2001; Touyz et al., 2002). In the years, since its discovery there has been considerable variation and controversy in identifying the principal location(s) of Nox4 in intact blood vessels. Using antisense riboprobes and a polyclonal antibody, Nox4 was identified primarily in the media of control rat carotid arteries and staining was most intense in the neointima of balloon injured vessels (Szocs et al., 2002). Similar staining patterns with robust staining of cells in the neointima have been observed in atherosclerotic human coronary arteries (Sorescu et al., 2002). Mittal et al. have shown, using both anti Nox4 antibodies and *in situ* hybridization, that Nox4 is primarily expressed in the medial layer of pulmonary blood vessels in both mice and humans (Mittal et al., 2007). In blood vessels, Nox4 is by far the most abundant Nox transcript (>1000 fold copy number over that of Nox1 and Nox2) (Matsuno et al., 2005). This is supported by studies in cultured cells which show that Nox4 mRNA is expressed at copy numbers greater than 10–1000 times that of Nox2 (Sorescu et al., 2002; Ago et al., 2004) and greater than 1000 times that of Nox1 (Dikalov et al., 2008). This data would suggest that Nox4 is the most abundant Nox isoform in the vasculature. However, one must be mindful that mRNA levels may not accurately reflect protein expression levels of the various Nox isoforms. In addition to VSMC (Touyz et al., 2002; Ellmark et al., 2005; Sturrock et al., 2006; Jay et al., 2008; Ismail et al., 2009) Nox4 is present in multiple cell types including endothelial cells and fibroblasts (Sorescu et al., 2002; Ago et al., 2004; Van Buul et al., 2005). In contrast to the early imaging studies, quantitative analysis of the relative level of Nox4 mRNA expression in cultured cells revealed that smooth muscle cells express proportionally, the least amount of Nox4, with higher levels found in endothelial cells and fibroblasts (Sorescu et al., 2002; Ago et al., 2004; Schroder et al., 2012). More recent studies using different Nox4 antibodies support this relationship and show that the majority of Nox4 staining in the intact aorta is confined to the endothelial cell layer (Craige et al., 2011; Ray et al., 2011). A caveat to these findings is that in intact blood vessels, the adventitial layer is frequently underrepresented or removed (Szocs et al., 2002). Adventitial staining has been observed in coronary blood vessels (Sorescu et al., 2002) and in rat aortic and human pulmonary fibroblasts (Haurani et al., 2008; Hecker et al., 2009; Amara et al., 2010). Nox4 has also been reported in immune cells such as macrophages (Lee et al., 2010) which can also populate the adventitia, particularly in inflammatory settings. The obligate Nox4 binding partner, p22phox has been shown to exhibit a similar pattern of expression and is present in all cell layers in intact blood vessels and cultured vascular cells (Ushio-Fukai et al., 1996; Azumi et al., 1999; Szocs et al., 2002). Nox4 expression has been reported in human and mouse cardiac myocytes (Byrne et al., 2003; Ago et al., 2010; Kuroda et al., 2010; Zhang et al., 2010). The relative abundance of Nox4 in the cardiac myocyte vs. vascular tissue is not well defined. In sum, there a wealth of evidence that Nox4 is expressed in all layers of the blood vessel and indeed all vascular and some perivascular cell types. However, the abundance and location of Nox4 in the respective cell types is likely to vary and be influenced by factors such as the organism in question, intramural pressure, inflammation, growth factors, oxygen concentration, and the type of vascular bed. A greater appreciation of how Nox4 is contributing to cardiovascular function in conditions of health and disease will need to be derived from imaging studies using well validated tools and cellular markers.

## Regulation of the expression and activity of Nox4

A number of studies have shown that Nox4 is robustly upregulated in response to transforming growth factor-beta (TGF-β) stimulation in a variety of cell types including aortic and pulmonary smooth muscle cells, pulmonary and cardiac fibroblasts, endothelial and embryonic kidney cells (Cucoranu et al., 2005; Hu et al., 2005; Sturrock et al., 2006; Hecker et al., 2009; Ismail et al., 2009; Xiao et al., 2009). Angiotensin II has been shown to potently activate Nox1 and Nox2 and subunits in vascular cells (Rajagopalan et al., 1996; Ushio-Fukai et al., 1996; Pagano et al., 1997; Touyz et al., 2002; Matsuno et al., 2005), but the effects on Nox4 expression are much less pronounced and perhaps indirect (Lassegue et al., 2001; Mollnau et al., 2002; Byrne et al., 2003; Cucoranu et al., 2005). These data suggest that vasoactive factors that influence blood vessel function in distinct ways can couple to specific Nox enzymes. Tumor necrosis factor-alpha is less specific and can increase both Nox1, 2 and Nox4 activity and or expression in a variety of vascular cells (Anilkumar et al., 2008; Basuroy et al., 2009; Moe et al., 2011). Other stimuli that induce Nox4 expression are endoplasmic reticulum (ER) stress (Pedruzzi et al., 2004), shear stress (Hwang et al., 2003), carotid artery balloon injury (Szocs et al., 2002), hypoxia and ischemia (Vallet et al., 2005; Lu et al., 2010), and the activation of PKCα (Xu et al., 2008). The down regulation of Nox4 expression has also been reported in response to platelet-derived growth factor (PDGF) and peroxisome proliferator-activated receptor-gamma (PPAR-gamma) ligands (Lassegue et al., 2001; Hwang et al., 2005), bone morphogenic protein 4 (BMP4) (Lambeth et al., 2007) and serum starvation of cultured cells (Peshavariya et al., 2009). A number of transcription factors have been shown to regulate Nox4 promoter activity including NFkB (Lu et al., 2010); SMAD2/3 (Sturrock et al., 2006), E2F (Zhang et al., 2008), HIF1α (Diebold et al., 2010), and Nrf2 (Pendyala et al., 2011) and these pathways are also likely dependent on the stimulus and cell type. Histone deacetylases can influence Nox4 expression by regulating transcription factor binding and miRNAs can influence mRNA levels at the posttranscriptional level (Pendyala et al., 2011; Siuda et al., 2012). A major limitation in our understanding of how Nox4 expression is regulated has been the lack and limited availability of specific Nox4 antibodies. A number of companies and individual laboratories have generated antibodies to Nox4 with surprisingly limited success (Zhang et al., 2011; Altenhofer et al., 2012). Given the variety of epitopes used, it is unlikely that antigenicity is the only barrier to success. More likely explanations include detergent insolubility (Jagnandan et al., 2007) and the tendency of transmembrane proteins to form aggregates (Prive, 2007). Therefore, studies using parallel approaches of real time PCR and Western blots have provided the most convincing data.

While Nox4 is regarded as being constitutively active and regulated in an inducible manner analogous to the inducible nitric oxide synthase (iNOS), there are several proteins that have been shown to modulate its activity. The best described of these is p22phox. Loss of p22phox expression abrogates Nox4 activity, without significant changes in its expression level. Increased expression of p22phox can also stimulate increased Nox4 activity. Poldip2 (polymerase DNA-directed delta-interacting protein 2) is a recently described protein that associates with p22phox to increase Nox4 activity. The mechanism by which Poldip2 regulates Nox4 activity is complex and involves not only the direct binding to p22phox, but increased targeting of Nox4 to cytoskeletal regions (Lyle et al., 2009). The toll like receptor 4 (TLR4) was found by yeast two hybrid and GST pull-down assays to interact with Nox4. The C-terminal region of Nox4 binds to the cytosolic region of TLR4 and mediates LPS-induced ROS production (Park et al., 2004, 2006). Other post-translational modifications of Nox4 are less well understood. In cells transfected with Nox4, multiple bands have been detected on Western blots, and it has been postulated that the larger band maybe result from glycosylation of Nox4. Four putative sites of glycosylation have been identified in the second and third extracellular loops of Nox4. However, digestion with glycosidase did not support glycosylation (Shiose et al., 2001). More recent reports suggest that some of the splice variants of Nox4 are glycosylated (Goyal et al., 2005), however, definitive evidence on whether endogenously expressed Nox4 or its variants are glycosylated is needed. Recently, we reported that Small Ubiquitin-like Modifier (SUMO1) was effective at suppressing Nox4 derived H_2_O_2_, but evidence of direct SUMOylation of Nox enzymes was not observed suggesting this is not a direct interaction (Pandey et al., 2011a). Protein methylation inhibitors did not influence Nox4-derived ROS production and the activity of Nox1 and 5, suggesting that protein methylation does not regulate the activity of NADPH-oxidases (Chen and Fulton, 2010). In native cells, the acute stimulation of Nox4 has been shown in response to the PKC agonist PMA in endothelial cells (Kuroda et al., 2005), angiotensin II in renal mesangial and tubule cells (Gorin et al., 2003; Kim et al., 2012), insulin in adipocytes (Mahadev et al., 2004), and IGF-1 in VSMC (Xi et al., 2012). This data is contrasted by results in more controlled heterologous expression systems, where Nox4 has been shown to be constitutively active and refractory to PMA, agonist stimulation and other Nox subunits (with the exception of p22phox) (Serrander et al., 2007; Helmcke et al., 2009; Nisimoto et al., 2010; von Lohneysen et al., 2010; Chen et al., 2011). The direct phosphorylation of Nox4 has not yet been described but remains a possibility given that other Nox isoforms are directly phosphorylated (Jagnandan et al., 2007; Pandey and Fulton, 2011; Pandey et al., 2011b). Indeed, analysis of Nox4 amino acid sequence (NetPhos2, GPS) reveals the presence of numerous PKC phosphorylation sites. Future studies will need to reconcile these observations and to determine whether Nox4 is acutely responsive to agonists in native cells and if so what factors are missing in the heterologous expression systems.

## Subcellular localization of Nox4

The intracellular distribution localization of Nox4 has been reported to influence a variety of Nox4 functions including enzyme activity, the type of ROS emitted from cells and the activation of distinct downstream signaling pathways (Hilenski et al., 2004; Kuroda et al., 2005; Serrander et al., 2007; Weyemi et al., 2010). While this has generated significant interest in the subcellular location of Nox4, it should not be surprising that this has also been another area of disparity. Nox4 has been reported in the nucleus of monocytes, endothelial and VSMC (Hilenski et al., 2004; Ago et al., 2005; Pendyala et al., 2009; Lee et al., 2010), focal adhesions in VSMC (Hilenski et al., 2004), the ER of transfected HEK, COS cells, thyroid, endothelial cells and VSMC (Ambasta et al., 2004; Van Buul et al., 2005; Martyn et al., 2006; Serrander et al., 2007; Chen et al., 2008; Helmcke et al., 2009; von Lohneysen et al., 2010; Weyemi et al., 2010; Wu et al., 2010), ER and nucleus of smooth muscle cells and renal cortical cells (Sturrock et al., 2007; Pendergrass et al., 2009), plasma membrane (PM) of lung epithelial cells and transfected COS (von Lohneysen et al., 2008, 2010) and the mitochondria of mesangial and renal cortical cells, cardiac myocytes and cancer cells (Block et al., 2009; Ago et al., 2010; Graham et al., 2010; Kuroda et al., 2010). The reasons for the different subcellular locations of Nox4 are likely to be manifold. Cell type could be a factor, but the mechanisms responsible for the cell type-specific targeting are unknown and it remains unclear why motifs such as a putative mitochondrial localization sequence (Graham et al., 2010) do not elicit mitochondrial targeting in all cell types. Nox4 has been shown to transition from one intracellular compartment to another and thus it is possible that some locations are transitory (Lyle et al., 2009; von Lohneysen et al., 2010). Alternatively, some splice variants of Nox4 have been reported to reside in the ER and others in the nucleus (Goyal et al., 2005). This may explain the presence of Nox4 in multiple intracellular compartments and the variation between cell types. However, the relative abundance of these variants in different cells is not known. Studies with fluorescent fusion proteins in live cells have consistently revealed ER or PM staining (Ambasta et al., 2004; von Lohneysen et al., 2010). Consistent with the results from live cell experiments, epitope tagged forms of Nox4 and NBT-staining of Nox4 in transfected cells have also revealed an endomembrane/ER location (Serrander et al., 2007; Chen et al., 2008; Helmcke et al., 2009). The live cell approach is devoid of potential antibody problems or fixation/permeabilization artifacts but like epitope tagging, is also dependent on the proper folding and targeting of the chimeric transgene and limited to cell types amenable to transfection. Caution should be exercised as the erroneous assignment of proteins to the nuclear or mitochondrial compartments has occurred for other proteins that are clearly not present or functional in these organelles (Jagnandan et al., 2005). The underlying reasons for the diversity of intracellular locations may relate to important cell-specific differences in the function of Nox4 or it may simply be due to poorly selective antibodies and or staining approaches. The development of a well vetted, commercially available Nox4 antibody would aid in this endeavor significantly.

## Nox4 signaling pathways and regulation of cellular function

Nox4 has been reported to activate a number of kinases including p38MAPK, Ras/ERK, JNK, and Akt (Djordjevic et al., 2005; Wu et al., 2007; Chen et al., 2008; Goettsch et al., 2009; Jaulmes et al., 2009). This may be due, at least in part, to the ability of Nox4-derived H_2_O_2_ to oxidize the catalytic cysteine residues and inhibit tyrosine phosphatases (Chen et al., 2008; Loh et al., 2009). In unstimulated HEK cells, Nox4 can activate Erk1/2 and JNK signaling pathways and Akt and p38 MAPK in response to challenge with insulin (Anilkumar et al., 2008). These data suggest that Nox4-dervived ROS can influence distinct signaling pathways that depend on the presence or absence of agonists. In VSMC, Nox4 can activate the small GTPase Rho (Brown and Griendling, 2009) which may be secondary to its ability to bind Poldip2 (Lyle et al., 2009). Nox4 expression and H_2_O_2_ play important roles in mediating oxygen sensing (Shiose et al., 2001), cell proliferation (Petry et al., 2006), differentiation (Clempus et al., 2007), migration (Lyle et al., 2009), apoptosis (Pedruzzi et al., 2004), senescence (Geiszt et al., 2000), matrix secretion (Hecker et al., 2009), and angiogenesis (Craige et al., 2011). The ability of Nox4 to regulate specific signaling pathways and cellular function appears to be dependent on the level of Nox4 expression, the intracellular location and the cell type. Given the high level of expression of Nox4 in vascular cell and its proposed role in multiple cellular processes, a surprising observation was that the genetic knockout of Nox4 did not result in an obvious baseline phenotype (Kleinschnitz et al., 2010; Schroder et al., 2012). Blood pressure (both systemic and pulmonary), cardiac function, endothelium-dependent relaxation, cerebral blood flow, and kidney function were unchanged in knockout mice.

## The role of Nox4 in cardiovascular diseases

Nox4 is expressed to varying degrees in virtually all cardiovascular cell types and can, at least in culture, significantly impact cellular function. In numerous cardiovascular diseases, such as hypertension, atherosclerosis, pulmonary and cardiac fibrosis, cardiac failure, stroke and diabetes, the expression level of Nox4 is elevated. As Nox4 is constitutively active and regarded as an inducible Nox or iNOX (Serrander et al., 2007), changes in expression suggest changes in ROS levels. Ideally, both mRNA and proteins levels should be assessed as similar to iNOS, it has been reported that Nox4 mRNA levels may not accurately reflect that of Nox4 protein (Peshavariya et al., 2009). The following subsections will outline current knowledge of the role of Nox4 in various cardiovascular diseases. A summary of the literature is included in Table 1.

**Table 1:** **Changes in Nox4 mRNA and protein expression and the effect of targeting Nox4 on cardiovascular diseases**.

**Disease**	**Organism**	**Nox4 mRNA**	**Nox4 Protein**	**Intervention**	**Effect on disease**	**References**
Cardiac failure	Human	↑-	–	–	–	Dworakowski et al., 2008; Borchi et al., 2010
Cardiac failure	Mouse	–	↑	Knockout	↓	Ago et al., 2010
Cardiac failure	Mouse	↑ -	–	Knockout	↑	Byrne et al., 2003; Zhang et al., 2010
Atrial fibrillation	Human	↑	–	None	–	Zhang et al., 2012
Stroke	Mouse	↑	↑	Knockout, Nox4 inhibitor	↓	Kleinschnitz et al., 2010
Pulmonary fibrosis	Mouse	↑	↑	Knockout, siRNA, Nox4 inhibitor	↓	Hecker et al., 2009; Carnesecchi et al., 2011
Pulmonary fibrosis	Human	↑	↑	None	–	Hecker et al., 2009; Amara et al., 2010
Hypertension	Rat	↑↓^*^	–	None	–	Nishiyama et al., 2004; Paravicini et al., 2004; Wind et al., 2010
Atherosclerosis	Human	↑	↑	None	–	Sorescu et al., 2002
Atherosclerosis	Mouse	–	–	None	–	Judkins et al., 2010
Diabetic renal injury	Mouse	–	↓	Knockout	↑	Babelova et al., 2012
Diabetic renal injury	Mouse	–	↑	Nox4 inhibitor	↓ Fibrosis	Sedeek et al., 2010
Pulmonary hypertension	Human	↑	↑	None	–	Mittal et al., 2007; Li et al., 2008
Pulmonary hypertension	Mouse	↑	↑	None	–	Mittal et al., 2007; Li et al., 2008

* Vessel-dependent.

### Hypertension

Elevated blood pressure has long been associated with increased ROS production in vascular tissue (Rajagopalan et al., 1996). The abundant expression of Nox4 in vascular and renal cells has led to numerous studies investigating its role in the elevation of blood pressure. However, the importance of Nox4 in hypertension remains unclear. In cultured VSMC, the pressor hormone, Angiotensin II increases Nox1 but not Nox4 expression (Lassegue et al., 2001) and cyclic strain, which can mimic increased blood pressure, decreases Nox4 in endothelial cells (Goettsch et al., 2009). In animal models, Nox4 levels have been reported to be decreased in the aorta of spontaneously hypertensive rats (SHR) (Wind et al., 2010). In contrast, others have shown that Nox4 mRNA expression is 4.1-fold higher in basilar arteries from SHR compared to normotensive Wistar-Kyoto rats (WKY) and in the renal cortex of DOCA-salt rats (Nishiyama et al., 2004; Paravicini et al., 2004). Thus, it is likely that the type of experimental hypertension and the location of the blood vessel studied can significantly impact how Nox4 expression is regulated. In the absence of pathogenic stimuli, Nox4-knockout mice do not have an obvious phenotype and are normotensive (Takac et al., 2012). Furthermore, transgenic overexpression of Nox4 in the vascular endothelium of mice lowers blood pressure (Ray et al., 2011). An interesting observation in this study was that despite the obvious higher level of Nox4 expression, endothelium-dependent relaxation was enhanced. This supports previous data showing that Nox4 emits primarily H_2_O_2_ instead of O^·−^_2_. A number of reports have also shown that Nox4 can stimulate eNOS-derived nitric oxide (NO^·^) (Craige et al., 2011; Schroder et al., 2012). The increased endothelial expression of Nox5 which produces both detectable O^·−^_2_ and H_2_O_2_ (Serrander et al., 2007), also resulted in increased eNOS activity, but in marked contrast to Nox4, there was significantly decreased biological effects of NO^·^ due to the interaction of NO^·^ and O^·−^_2_ (Zhang et al., 2008). A complication in the interpretation of overexpression strategies is that the level of Nox4 and attendant ROS production is an important variable and chronically high levels may stimulate feedback inhibition and limit the endogenous expression of Nox4 with unpredictable consequences on cellular function (Haurani et al., 2008).

### Atherosclerosis

Multiple Nox enzymes have been implicated in the development of atherosclerotic plaques (Madamanchi and Runge, 2010). In human atherosclerosis, Nox4 expression is increased in intimal lesions of coronary arteries (Sorescu et al., 2002). However, in experimental atherosclerosis, Nox4 protein levels are unchanged in the aorta of genetically susceptible mice (Judkins et al., 2010) or in primate models (Stanic et al., 2012). However, there is much evidence in cell culture to suggest that Nox4 may be involved in atherogenesis. Laminar flow which has been shown to be protective against atherosclerosis actually decreases Nox4 expression (Goettsch et al., 2011) whereas oscillatory shear stress increases it (Hwang et al., 2003). Moreover, a variety of oxidized lipids can stimulate Nox4 expression. Oxidized LDL increases Nox4 expression in macrophages (Lee et al., 2009), 7-ketocholesterol stimulates Nox4 expression in VSMC to induce ER stress (Pedruzzi et al., 2004) and in endothelial cells, 1-palmitoyl-2-arachidonyl-sn-glycerol-3-phosphocholine (Ox-PAPC) induces Nox4 expression (Lee et al., 2009). Despite these observations, a clear role for Nox4 in atherosclerosis remains to be determined. It is likely that Nox4 expression is influenced by a multitude of factors including the severity of the lesion (Sorescu et al., 2002), the location of the blood vessel and individual factors such as other concurrent diseases such as inflammation and heart failure (Guzik et al., 2006). More definitive studies are needed, particularly with regard to the characterization of Nox4 expression in lesions of human blood vessels and in animal models of atherosclerosis such as the diabetic pig (Gerrity et al., 2001) that may better emulate advanced human atherosclerotic lesions using genetic or pharmacological inhibition of Nox4.

### Pulmonary arterial hypertension

Pulmonary Arterial Hypertension (PAH) arises from the narrowing of pulmonary arteries which elevates pulmonary vascular resistance and consequently pulmonary artery blood pressure. The chronic elevation of pulmonary vascular resistance is secondary to occlusive remodeling of pulmonary blood vessels (Humbert et al., 2004; Cool et al., 2005). PAH is characterized by excessive proliferation and hypertrophy of pulmonary arterial medial smooth muscle (Sturrock et al., 2006), adventitial remodeling (Stenmark et al., 2006) and the presence of plexiform lesions (Tuder et al., 1994). ROS are important regulators of pulmonary vascular remodeling, and abundant evidence supports a prominent role for Nox4 in the pathogenesis of PAH (Mittal et al., 2007; Nisbet et al., 2009). Nox4 is the major NADPH oxidase homolog expressed in human pulmonary artery smooth muscle cells (Sturrock et al., 2006), and its expression both at the mRNA and protein level is significantly increased in lungs from patients with idiopathic pulmonary arterial hypertension (IPAH) compared to healthy lungs (Mittal et al., 2007), which suggests a correlation between Nox4 and the onset of PAH.

In experimental models of PAH in rodents, Nox4 expression is also increased in chronic hypoxia-induced PAH in mice (Mittal et al., 2007; Li et al., 2008; Nisbet et al., 2009) and monocrotaline in rats (Dorfmuller et al., 2011). Nox4 mediates the hypoxia-induced growth of human pulmonary smooth muscle cells (Ismail et al., 2009), and silencing Nox4 expression by RNA interference decreases human pulmonary arterial smooth muscle cell and fibroblast proliferation (Sanders and Hoidal, 2007; Li et al., 2008; Griffith et al., 2009). Nox4 has been shown to contribute to angiogenesis (Craige et al., 2011; Schroder et al., 2012) and mediates the induction of VEGF and angiogenesis in response to pressure overload in the heart (Zhang et al., 2010). Severe forms of PAH are associated with plexiform lesions which are comprised of proliferating endothelial cells and elevated levels of angiogenic factors such as VEGF (Tuder et al., 2001; Jonigk et al., 2011). Pneumonectomy increases the severity of PAH in animals treated with monocrotaline and has been shown to stimulate the formation of lesions that are morphologically similar to plexiform lesions (Bauer et al., 2007). Pneumonectomy also further increases the expression of Nox4 in monocrotaline-treated animals (Dorfmuller et al., 2011), but it is not yet known whether Nox4 is expressed in or contributes to the formation of these lesions. Collectively, these findings support the argument for a role of Nox4 in medial smooth muscle proliferation, endothelial proliferation and adventitial fibroblast-activation in PAH. Nox4 expression has been shown in the media of both normotensive and hypertensive pulmonary arteries (Mittal et al., 2007). This data is consistent with earlier reports of the location of Nox4 in systemic arteries but contrasts from reports of higher expression levels of Nox4 in endothelial cells and fibroblasts (Sorescu et al., 2002; Ago et al., 2004; Schroder et al., 2012). This may reflect the unique environment of the pulmonary circulation but warrants additional investigation. Pulmonary blood pressure and right ventricular systolic pressure in Nox4 knockout mice is unchanged under normoxic conditions and hypoxic vasoconstriction *in vitro* is equivalent to control mice (Kleinschnitz et al., 2010). Whether pulmonary hypertension and remodeling of pulmonary arterioles and the right ventricle in response to chronic hypoxia is altered in the Nox4 knockout mouse is not yet known. It would also be important to assess a role for Nox4 in additional models such as the rat which develop a more robust pulmonary hypertension that is closer to the human condition.

### Fibrosis

Nox4 is expressed in fibroblasts and expression is very strongly induced by TGF-beta (Cucoranu et al., 2005; Hecker et al., 2009). Hydrogen peroxide is a well-established pro-proliferative stimulus (Davies, 1999) and Nox4, is the only Nox isoform that preferentially emits H_2_O_2_. Nox4 expression in fibroblasts promotes changes in proliferation, differentiation, migration, contractility, and extracellular matrix secretion (Crestani et al., 2011). Nox4 expression is robustly increased in pulmonary fibroblasts from patients with idiopathic pulmonary fibrosis and also in rodent models (Hecker et al., 2009; Amara et al., 2010). Inhibition of Nox4 using genetic or pharmacological approaches prevents lung fibrosis (Hecker et al., 2009; Amara et al., 2010; Carnesecchi et al., 2011) and suggests a prominent role for Nox4 in the pathogenesis of pulmonary fibrosis. However, others have reported that some of the Nox4 knockout mice do not provide protection against fibrosis (Altenhofer et al., 2012). An explanation for these divergent findings has not been advanced and will likely only be settled with additional studies using better tools. For example, it has been reported that there are significant differences in the Nox4 knockout models with regard to the targeting strategy and whether Nox4 expression and activity is fully lost (Altenhofer et al., 2012). The loss of Nox4 does not result in the upregulation of other Nox proteins, but the Nox enzymes are only one of many sources of cellular ROS. It remains to be determined how the developmental loss of Nox4 impacts other sources of ROS such as mitochondria, eNOS and other enzymes. More elegant studies using inducible strategies in floxed mice would also be advantageous to avoid developmental adaptation. The same advantage would be derived from selective inhibitors of Nox4 with the added benefit that it could be employed in additional organisms.

### Cardiac failure

Nox4 is induced in experimental models of heart failure (Byrne et al., 2003; Ago et al., 2010) and in humans (Dworakowski et al., 2008). Recent studies using cardiac specific Nox4 knockout mice reveal decreased levels of ROS and improved performance along with reduced hypertrophy, fibrosis and apoptosis. Gain of function experiments using a transgenic cardiac specific Nox4-overexpressing mouse had the opposite phenotype, promoting dysfunction, fibrosis, and apoptosis in response to pressure overload (Kuroda et al., 2010). While these results suggest that Nox4 is a major source of oxidative stress involved in the failing heart and is deleterious, others have shown the opposite results using a global Nox4 knockout and a cardiac specific Nox4 transgenic (Zhang et al., 2010). A difference between these studies is the method used to induce heart failure with transverse aortic constriction of the ascending aorta used in the former and abdominal constriction (descending) used in the latter. Further studies are needed to determine how Nox4 may mediate such contradictory roles.

### Stroke

Elevated ROS have been linked to the severity of ischemic stroke (Gilgun-Sherki et al., 2002). Nox isoforms including Nox4 are expressed at much higher levels in cerebral vs. systemic blood vessels (Miller et al., 2005). Nox4 expression is upregulated in human and mouse brain post occlusion of the middle cerebral artery (Kleinschnitz et al., 2010). Genetic deletion and pharmacological inhibition of Nox4 reduced infarct volume, whereas genetic deletion of Nox1 and Nox2 and treatment with apocynin had no effect (Kleinschnitz et al., 2010). Therefore it is likely that Nox4 is an important mediator of ischemia stroke. However, others have shown that apocynin is effective against stroke and more specifically that Nox2 may be important in ischemic stroke (Jackman et al., 2009; Kahles and Brandes, 2012). While, there is much evidence to support a role for Nox4 in stroke and other diseases, controversy is unfortunately the most consistent theme throughout. A good analogy is the parable of the blind men and the elephant where unique and imperfect tools to study Nox4 lack the necessary vision to provide an accurate picture that all can agree on.

## Concluding remarks

In summary (see also Figure 1), there are three important aspects to our understanding of Nox4. The first is that, Nox4 is truly unique among the Nox isoforms. This is reflected by its high level of expression in vascular cells, constitutive activity, subcellular location, the pattern of ROS that it produces and its insensitivity to Hsp90 inhibitors. The second is that Nox4 is an inducible Nox or iNox and its expression level is dynamically regulated in response to a wide range of stimuli. The third is that we don't yet fully understand the physiological and pathophysiological roles of Nox4. Expression levels of Nox4 are increased in a number of diseases including fibrosis, PAH and stroke, but the functional role ascribed to Nox4 is frequently both deleterious and beneficial (Brandes et al., 2011; Altenhofer et al., 2012; Schmidt et al., 2012; Schroder et al., 2012). This is an important conundrum and one which can only be addressed properly with additional studies. In cell culture, where the variables can be more tightly controlled, there is a much clearer consensus on the functional role of Nox4. In contrast, in animals, there are many possibilities to account for the wide variation. These include the interaction of multiple cell types that express Nox4, differences between the various Nox4 knockout mice, developmental compensation for the loss of Nox4, a paucity of reliable inhibitors and the suitability of the mouse as a model of cardiovascular disease. The latter is particularly true with regard to PAH, where a number of rodent models are necessary to emulate specific aspects of the human disease. Mice are also inferior to rats for blood pressure research. Another consideration is the assumption that changes in Nox4 expression have a linear effect on cellular function. On the contrary, it is very likely that the amount of ROS produced by Nox4 can have varying effects with low levels of Nox4 expression having effects that are quite distinct from high levels. There has been recent discussion on whether Nox4 is good or bad and while this is understandable given the current state of ambiguity in our understanding of Nox4, it is also worth noting that there is little evolutionary pressure to retain genes whose only function is to cause disease. In contrast, the principles of toxicology are also worthy of consideration in this debate as too much of a good thing can also be bad. Certainly this is a well-established theme with regard to inflammation and even endothelial nitric oxide synthase. Future studies will be better served with improved tools including antibodies, genetic models and inhibitors and these will all be necessary to reveal the true nature of the role of Nox4 in both health and disease.

### Conflict of interest statement

The authors declare that the research was conducted in the absence of any commercial or financial relationships that could be construed as a potential conflict of interest.

## Abbreviations

VSMC: vascular smooth muscle cells
ER: endoplasmic reticulum
PM: plasma membrane
O^·−^_2_: superoxide
H_2_O_2_: hydrogen peroxide
NO: nitric oxide.
